# Effects of Berberine on Lipid Metabolism, Antioxidant Status, and Immune Response in Liver of Tilapia (*Oreochromis niloticus*) under a High-Fat Diet Feeding

**DOI:** 10.3390/antiox13050548

**Published:** 2024-04-29

**Authors:** Rui Jia, Yiran Hou, Liqiang Zhang, Bing Li, Jian Zhu

**Affiliations:** 1 Key Laboratory of Integrated Rice-Fish Farming Ecology, Ministry of Agriculture and Rural Affairs, Freshwater Fisheries Research Center, Chinese Academy of Fishery Sciences, Wuxi 214081, China; jiar@ffrc.cn (R.J.); houyr@ffrc.cn (Y.H.); zhangliqiang@ffrc.cn (L.Z.); 2 Wuxi Fisheries College, Nanjing Agricultural University, Wuxi 214081, China

**Keywords:** berberine, high-fat diet, lipid metabolism, liver damage, *Oreochromis niloticus*

## Abstract

Berberine, a natural alkaloid found abundantly in various medicinal plants, exhibits antioxidative, anti-inflammatory, and lipid metabolism-regulatory properties. Nonetheless, its protective effects and the molecular mechanisms underlying liver injury in fish have not been fully elucidated. The aims of this study were to investigate the antioxidative, anti-inflammatory, and lipid metabolism-regulating effects of berberine against high-fat diet (HFD)-induced liver damage and to clarify the underlying molecular mechanisms. Tilapia were fed diets containing two doses of berberine (50 and 100 mg/kg diet) alongside high fat for 60 days. The results showed that berberine treatments (50 and/or 100 mg/kg) significantly reduced elevated aminotransferases, triglycerides (TG), total cholesterol (TC), and low-density lipoprotein cholesterol (LDL-c) in the plasma. In the liver, berberine treatments significantly increased the expression of peroxisome proliferator-activated receptor α (*pparα*) and carnitine palmitoyltransferase 1 (*cpt-1*) genes, leading to a reduction in lipid accumulation. Meanwhile, berberine treatment suppressed lipid peroxidation formation and enhanced antioxidant capacity. Berberine upregulated the mRNA levels of erythroid 2-related factor 2 (*nrf2*) and its downstream genes including heme oxygenase 1 (*ho-1*) and glutathione-S-transferase (*gstα*). Additionally, berberine attenuated the inflammation by inhibiting the expression of toll-like receptor 2 (*tlr2*), myeloid differential protein-88 (*myd88*), *relb*, and inflammatory cytokines such as interleukin-1β (*il-1β*), tumor necrosis factor-α (*tnf-α*), and *il-8*. In summary, this study suggested that berberine offers protection against HFD-induced liver damage in tilapia via regulating lipid metabolism, antioxidant status, and immune response. This protective effect may be attributed to the modulation of the Nrf2, TLR2/MyD88/NF-κB, and PPARα signaling pathways.

## 1. Introduction

The liver is crucial in fish, serving as the central organ for metabolism of substances and energy and providing vital barrier functions through detoxification and phagocytosis. It is susceptible to damage due to a variety of factors, including exposure to heavy metals, misuse of chemical medications or antibiotics, and changes in environments [1,2]. Abnormal liver function can suppress growth, disrupt normal metabolism, reduce immunity and stress tolerance, and may even lead to death. In aquaculture, fatty liver is a common metabolic dysfunction disease of fish liver [3]. There are numerous inducing factors, such as nutritional imbalances, environmental stress, and abnormalities in physiological functions, all of which can lead to excessive lipid accumulation or lipid metabolic disorders in the liver of fish, thereby causing liver lesions [4]. The mechanism underlying fatty liver injury has been extensively reported in fish, implicating lipid accumulation, oxidative stress, and inflammatory responses [5,6]. Diets rich in fat have been found to disrupt lipid metabolism, exacerbate lipid peroxidation, and impair immune function in the liver of tilapia (*Oreochromis niloticus*) [7,8]. Similarly, a high-fat diet (HFD) has been observed to cause lipid deposition, oxidative stress, and chronic inflammation in the liver of blunt snout bream (*Megalobrama amblycephala*) [9,10].

Given the adverse impact of fatty liver on aquaculture fish, researchers have been exploring preventive and therapeutic measures. Du et al., (2013) have recommended employing nutritionally balanced diets, preventing feed deterioration, enhancing aquaculture technology, and mitigating stress in fish as viable strategies [11]. Alongside, the exploration of pharmacological agents, particularly herbal extracts with hepatoprotective and antioxidant properties, has opened new avenues for treating fatty liver disease in fish. Notably, dietary *Eucommia ulmoides* leaf extract alleviated liver steatosis and improved liver function in *Ictalurus punctatus* [12]. Similarly, resveratrol has been found to regulate lipid synthesis and metabolism in the liver of *O. niloticus*, thus reducing liver damage [13]. Saikosaponin d, by acting on the AMPK/PPARα pathway, has been effective in countering hepatic steatosis induced by an HFD in hybrid grouper (*Epinephelus lanceolatusd*♂ × *Epinephelus fuscoguttatus*♀) [14]. Additionally, dietary betaine has been shown to effectively mitigate hepatic inflammation induced by an HFD in *Acanthopagrus schlegelii* [15]. These findings clearly demonstrate the substantial potential of herbal extracts in improving liver health and combating lipid accumulation in fish. Therefore, integrating these natural compounds into aquaculture practices could serve as an effective strategy for mitigating fatty liver disease, ultimately enhancing the welfare and productivity of cultured fish.

Berberine, a natural alkaloid, is prevalent in numerous medicinal plants, especially in traditional Chinese medicinal species like *Coptis chinensis*, *Berberis vulgaris*, and *Hydrastis Canadensis* [16]. Recent studies highlighted berberine’s extensive potential in medicine, especially as a treatment option for diabetes, cardiovascular disease, fatty liver, and specific chronic inflammatory disorders [17]. Its capacity to regulate metabolic pathways, decrease blood glucose and cholesterol levels, alongside its antioxidant capabilities, further validate berberine’s therapeutic promise [18]. In aquaculture, berberine has been investigated as a dietary supplement, demonstrating beneficial effects in various fish species [19]. Dietary berberine was reported to promote growth and decrease the mortality of *M. amblycephala* induced by *Aeromonas hydrophila* exposure [20]. Yu et al. (2020) found that berberine improved intestinal barrier function by modulating the intestinal microbiota in *M. amblycephala* [21]. Furthermore, it has been demonstrated that berberine could promote lipid metabolism and enhance antioxidant capacity in *Mylopharyngodon piceus* [22]. In studies focusing on liver health, berberine has shown its effectiveness by reducing hepatocyte apoptosis induced by an HFD in *M. amblycephala* [23] and by alleviating chronic liver injury induced by copper exposure in *Acrossocheilus fasciatus* [24]. These findings highlight the necessity for further investigations into the impact of berberine on liver functions across a broader spectrum of fish species. Conducting such extensive research is crucial to deepen our understanding of berberine’s therapeutic potential and its possible application in aquaculture.

Tilapia (*O. niloticus*) is extensively cultured in regions including China, Asia, and Africa. In 2022, the production of tilapia in China reached 1,738,947 tons. In intensive tilapia aquaculture, the common practice of overfeeding or providing diets high in fats and sugars to accelerate growth frequently leads to the emergence of fatty liver disease. HFD has been confirmed to induce oxidative stress, disrupt lipid metabolism, reduce immune capacity, and damage liver tissue in tilapia [25,26,27]. Several extracts from traditional Chinese herbs, such as resveratrol [13], total flavanones from *Sedum sarmentosum Bunge* [28], and total flavones from *Glycyrrhiza* [29], have been found to regulate lipid metabolism, suppress oxidative stress, alleviate inflammation, and consequently ameliorate HFD-induced liver damage in tilapia. However, there is a notable absence of research on the protective effects and the underlying molecular mechanisms of berberine against fatty liver damage in tilapia. Therefore, it is interesting to investigate the protective effects of berberine using HFD-induced liver damage model in tilapia, focusing on its impacts on lipid metabolism, oxidative stress, and immune responses.

## 2. Materials and Methods

### 2.1. Tilapia, Experimental Design, and Sampling

Juvenile tilapia, weighing 52 ± 2.2 g, were obtained from the Freshwater Fish Research Center of the Chinese Academy of Fishery Sciences (Wuxi, China) and underwent a two-week acclimatization to laboratory conditions in a recirculation system, which maintained a temperature of 29 ± 2 °C, dissolved oxygen levels above 6 mg/L, and a pH range of 7.4–7.9. Prior to initiating the experiment, these fish were fed a control diet twice daily.

Post-acclimatization, the tilapia were weighed and systematically allocated into four distinct groups: a normal control group (NC), a high-fat diet group (HFD), and two groups receiving 50 mg/kg and 100 mg/kg of berberine, respectively. The NC group received a control diet consisting of 6% fat, while the HFD group was fed a high-fat diet containing 21% fat. The berberine-supplemented groups were fed diets containing either 50 mg/kg or 100 mg/kg of berberine, complemented by 21% fat. The formulation of the high-fat diet was based on methodologies validated in previous studies [30,31]. The inclusion rate of berberine in the diets was selected according to previous studies [32,33]. Each group consisted of 60 fish, tested across three replicates. The fish were fed approximately 4% of their body weight twice daily (09:00 and 16:00), over a period of 60 days.

After 60 days of feeding, the tilapia were weighed, and nine fish from each group were randomly selected for the collection of liver and blood tissues, conducted under anesthesia using 100 mg/L MS-222 (Sigma-Aldrich, Shanghai, China). The plasma was isolated from the blood via centrifugation at 5000 rpm, 4 °C, for 10 min, facilitating the analysis of blood biochemical parameters. Liver samples were immediately flash-frozen in liquid nitrogen to preserve them for subsequent assessments of enzymatic activity and gene expression.

### 2.2. Biochemical Parameter Analysis

Plasma biochemical parameters, including total triacylglycerol (TG), total cholesterol (TC), low-density lipoprotein cholesterol (LDL-c), high-density lipoprotein cholesterol (HDL-c), glutamate pyruvate transaminase (GPT), glutamate oxaloacetate transaminase (GOT), total protein (TP), albumin (Alb), alkaline phosphatase (AKP), and acid phosphatase (ACP), were quantified using commercial assay kits. These measurements followed the protocols provided by the Nanjing Jiancheng Bioengineering Institute (Nanjing, China).

Antioxidative parameters, including superoxide dismutase (SOD), total antioxidant capacity (T-AOC), glutathione (GSH), and malondialdehyde (MDA), were measured in liver samples utilizing commercial assay kits, following the protocols specified by the manufacturer (Nanjing Jiancheng Bioengineering Institute, Nanjing, China).

### 2.3. Measurement of Target Gene Expression

RNA was extracted from 100 mg of tilapia liver tissue employing RNAiso Plus (Takara, Beijing, China), chloroform, isopropanol, and ethanol. Spectrophotometric analysis measured the OD_260/280_ values to assess RNA quality and concentration. The PrimeScript™ RT Reagent Kit with gDNA Eraser (Takara) was utilized to convert RNA into first-strand cDNA via a two-step reverse transcription process. This cDNA was then used as a template for quantitative real-time PCR (qPCR). The qPCR reactions utilized TB Green^TM^ Premix EX Taq^TM^ II (Takara) in a total volume of 25 μL, comprising 12.5 μL TB Green^TM^ Premix EX Taq^TM^ II, 1 μL each of forward and reverse primers, 8.5 μL of ddH2O, and 2 μL of cDNA. For normalization of gene expression, the ubiquitin-conjugating enzyme (*ucbe*) was utilized as a reference gene. The relative mRNA levels were determined using the 2^−ΔΔCq^ method [34]. The specific primers used for qPCR in tilapia are detailed in Table 1.

### 2.4. Statistical Analysis

In this study, data were processed using SPSS 24.0 for analysis and GraphPad Prism 5 software for visualization. Analyses for normal distribution and homogeneity of variance were performed on all data. Differences between groups were determined using one-way analysis of variance (ANOVA), followed by the LSD test for instances of equal variances and Tamhane’s T2 test for cases of unequal variances, with significance established at *p* < 0.05. Results are expressed as mean ± standard error of the mean (mean ± SEM).

## 3. Results

### 3.1. Changes in Hepatic Damage Parameters in Plasma

**Figure 1:**
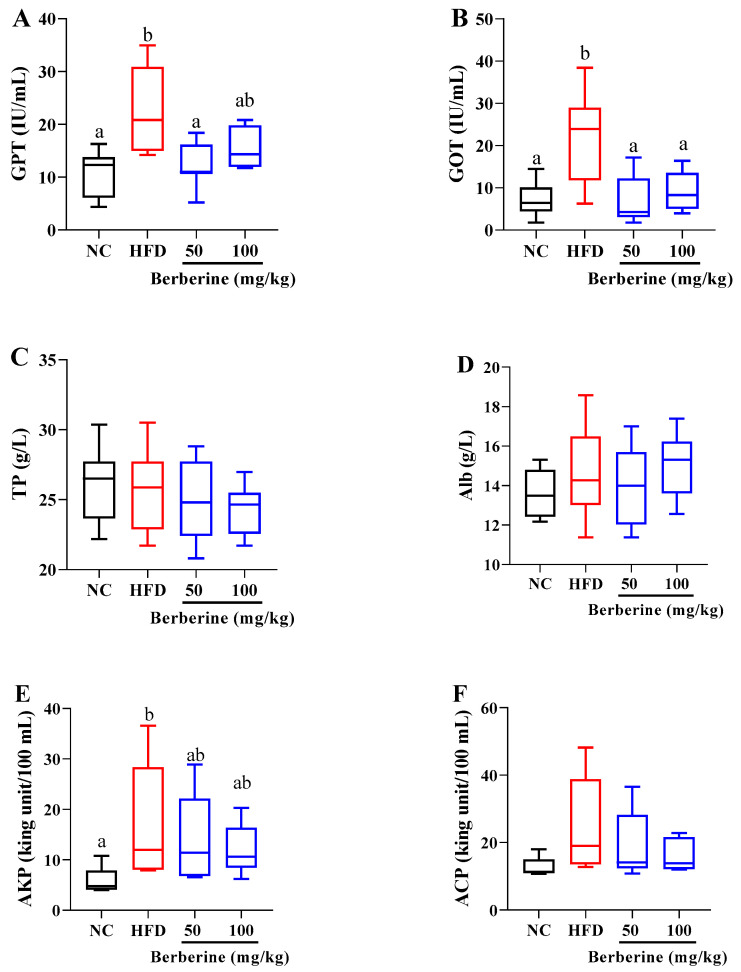
Changes in plasma hepatic damage parameters in tilapia fed berberine-inclusive high-fat diet. Different lowercase letters indicate significant differences between groups. (**A**) Glutamate pyruvate transaminase (GPT). (**B**) Glutamate oxaloacetate transaminase (GOT). (**C**) Total protein (TP) (**D**) Albumin (Alb). (**E**) Alkaline phosphatase (AKP). (**F**) Acid phosphatase (ACP).

In the plasma, HFD feeding alone significantly elevated the levels of GPT and GOT after 60 days. However, these alterations were significantly mitigated by treatment with berberine at the dose of 50 mg/kg (Figure 1A,B). Similarly, the increased GOT was markedly decreased in the group treated with 100 mg/kg berberine (Figure 1B). Moreover, the AKP level was elevated in the HFD group, whereas berberine treatment failed to mitigate this increase (Figure 1E). Additionally, the levels of TP, Alb, and ACP were unchanged by either the HFD or berberine treatment (Figure 1C,D,F).

### 3.2. Change in Lipid Metabolism in Plasma

**Figure 2:**
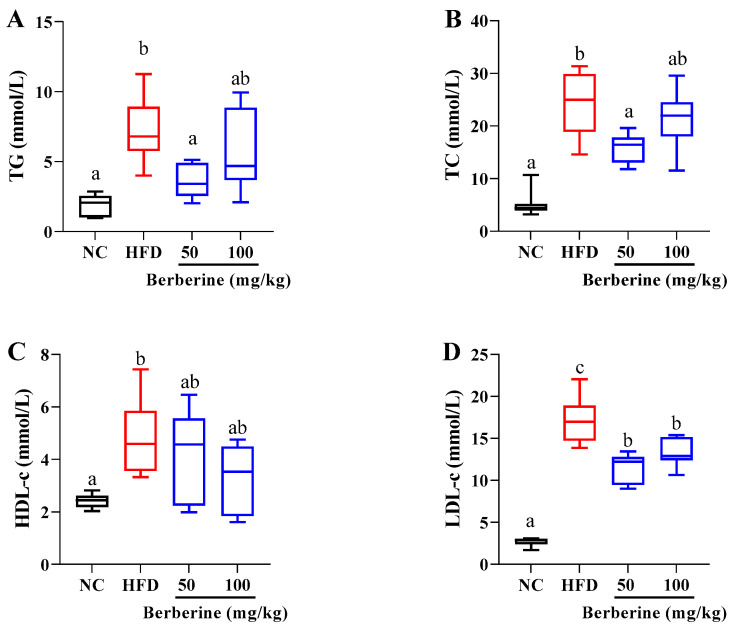
Changes in plasma lipid metabolism parameters in tilapia fed berberine-inclusive high-fat diet. Different lowercase letters indicate significant differences between groups. (**A**) Total triacylglycerol (TG). (**B**) Total cholesterol (TC). (**C**) Low-density lipoprotein cholesterol (LDL-c). (**D**) High-density lipoprotein cholesterol (HDL-c).

As shown in Figure 2, the plasma parameters’ analysis displayed that an HFD-alone treatment significantly raised the levels of TG, TC, LDL-c, and HDL-c after 60 days. Notably, the elevation in TG and TC levels was significantly ameliorated in the group receiving 50 mg/kg of berberine. Furthermore, berberine treatment at dosages of both 50 and 100 mg/kg markedly reduced the increases in LDL-c (*p* < 0.05). 

### 3.3. Changes in the Expression of Genes Related to Metabolism Function

**Figure 3:**
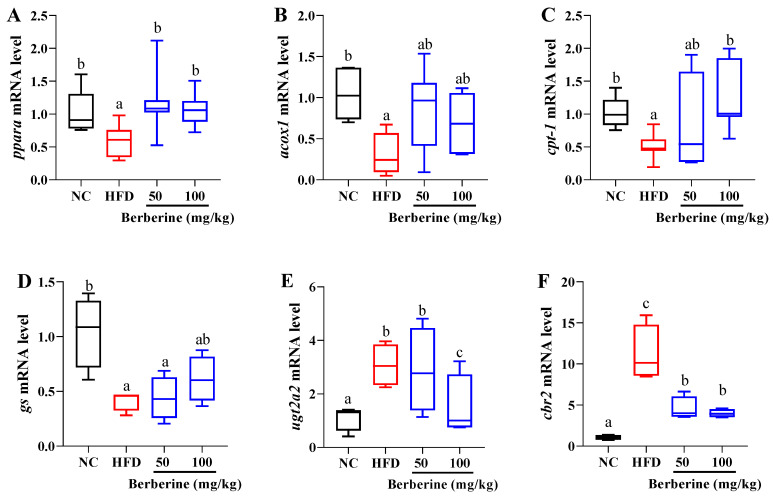
Relative expression of genes related to metabolism function in liver of tilapia fed berberine-inclusive high-fat diet. Different lowercase letters indicate significant differences between groups. (**A**) Peroxisome proliferator activated receptor alpha (*pparα*). (**B**) Acyl-CoA oxidase 1 (*acox1*). (**C**) Carnitine O-palmitoyltransferase 1 (*cpt-1*). (**D**) Glutamine synthase a (*gs*). (**E**) UDP-glucuronosyltransferase 2A2 (*ugt2a2*). (**F**) NADH-cytochrome b5 reductase 2 (*cbr2*).

To explore berberine’s role in regulating lipid metabolism, we examined the mRNA levels of fatty acid β-oxidation-related genes, including *pparα*, *acox1*, and *cpt-1* in the liver (Figure 3). The results highlighted a significant decrease in the expression of *pparα* in the group treated with an HFD in comparison to that in the NC group (*p* < 0.05; Figure 3A). However, this decrease was significantly reversed by berberine treatment with 50 and 100 mg/kg when compared to the HFD-only treatment (*p* < 0.05). The *cpt-1* mRNA level was significantly reduced in the HFD group; however, this downregulation was mitigated by treatments with 100 mg/kg of berberine (*p* < 0.05; Figure 3C). In addition, *acox1* mRNA level was notably lower in the HFD group, but the downregulation was not significantly counteracted by berberine treatments (Figure 3B).

To investigate the relationship between other metabolism function and the hepatoprotective effects of berberine against HFD-induced liver damage, we measured the mRNA levels of *gs*, *ugt2a2*, and *cbr2*. Compared with the NC group, HFD treatment led to an increase in the mRNA levels of *ugt2a2* and *cbr2*, while causing a decrease in *gs* mRNA level (*p* < 0.05; Figure 3D–F). The mRNA levels of *ugt2a2* and *cbr2* were significantly reduced following berberine treatment with 100 mg/kg compared with the HFD group (*p* < 0.05; Figure 3D,E). However, berberine treatment did not change the expression of *gs* compared with the HFD group (*p* > 0.05; Figure 3F).

### 3.4. Changes in Antioxidation Status in Liver

**Figure 4:**
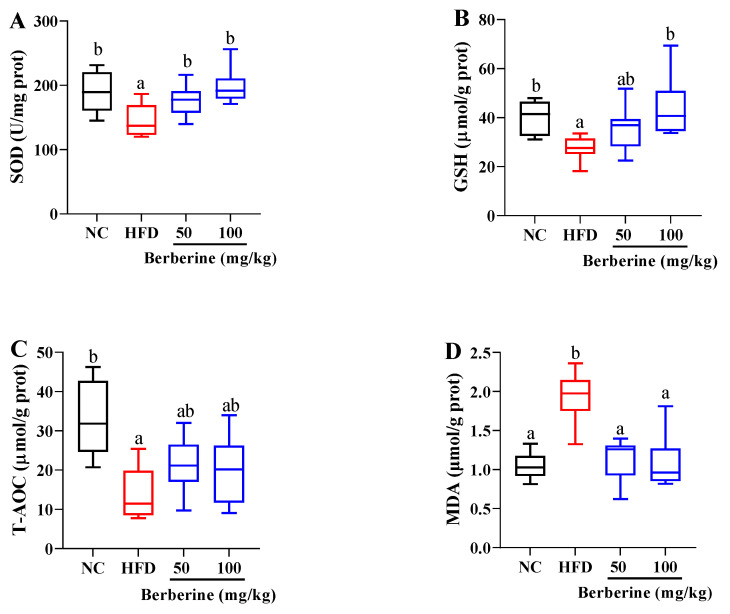
Changes in antioxidant status in liver of tilapia fed berberine-inclusive high-fat diet. Different lowercase letters indicate significant differences between groups. (**A**) Superoxide dismutase (SOD). (**B**) Glutathione (GSH). (**C**) Total antioxidant capacity (T-AOC). (**D**) Malondialdehyde (MDA).

In the liver, there was a marked reduction in the levels of SOD, GSH, and T-AOC, accompanied by an increase in the MDA level, in tilapia treated with an HFD alone, compared with those in the NC group (*p* < 0.05; Figure 4). The decrease in SOD level was markedly improved by berberine treatment at both 50 and 100 mg/kg (*p* < 0.05). Similarly, the reduction in GSH level was significantly ameliorated with berberine treatment at a dose of 100 mg/kg (*p* < 0.05). Furthermore, the elevation in the MDA level was substantially reduced under berberine treatment at both 50 and 100 mg/kg doses (*p* < 0.05).

### 3.5. Changes in the Expression of Genes Related to Antioxidant Status

**Figure 5:**
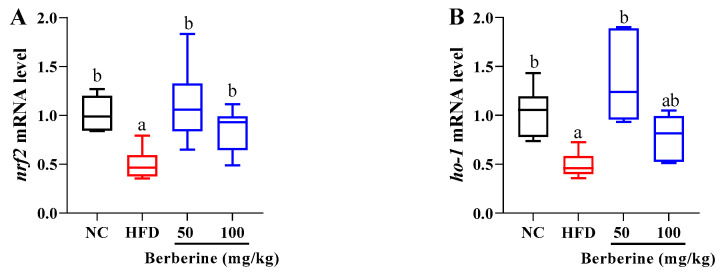

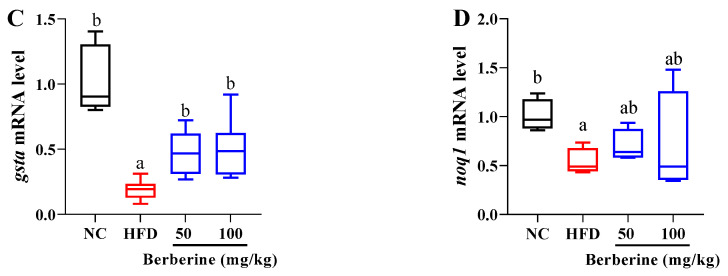
Relative expression of genes related to antioxidant status in liver of tilapia fed berberine-inclusive high-fat diet. Different lowercase letters indicate significant differences between groups. (**A**) Nuclear factor erythroid 2-related factor 2 (*nrf2*) (**B**) Heme oxygenase (*ho-1*). (**C**) Glutathione S-transferase (*gsta*). (**D**) NAD(P)H dehydrogenase 1 (*nqo1*).

After HFD feeding, the mRNA levels of *nrf2* and *gstα* were notably downregulated when compared with those in the NC group (*p* < 0.05; Figure 5A,C), while this downregulation was significantly alleviated in the groups treated with berberine at doses of 50 and 100 mg/kg (*p* < 0.05; Figure 5A,C). Similarly, the mRNA level of *ho-1* was markedly reduced after 60 days of HFD feeding, whereas this reduction was prevented by treatment with 100 mg/kg of berberine (*p* < 0.05; Figure 5B). In addition, the expression of *nqo1* was significantly reduced after HFD feeding, whereas berberine treatments did not influence the *nqo1* expression (*p* > 0.05; Figure 5D). 

### 3.6. Changes in the Expression of Genes Related to Inflammatory Response

**Figure 6:**
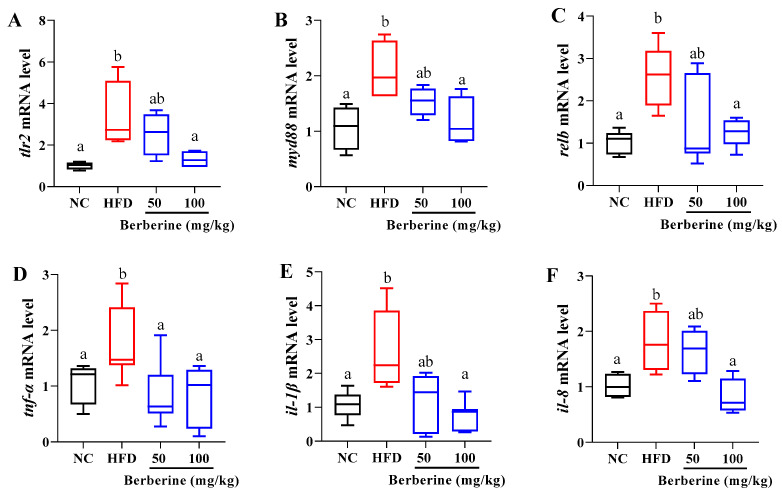
Relative expression of genes related to inflammatory response in liver of tilapia fed berberine-inclusive high-fat diet. Different lowercase letters indicate significant differences between groups. (**A**) Toll-like receptor 2 (*tlr2*). (**B**) Myloid differentiation factor 88 (*myd88*). (**C**) NF-kB subunit (*relb*). (**D**) Tumor necrosis factor-alpha (*tnf-α*). (**E**) Interleukin-1 beta (*il-1β*). (**F**) Interleukin-8 (*il-8*).

The expression of genes associated with the inflammatory response in the liver of tilapia are depicted in Figure 6. The mRNA levels of *tlr2*, *myd88*, *relb*, *tnf-α*, *il-1β*, and *il-8* were significantly elevated in the HFD group compared to those in the NC group (*p* < 0.05). These genes exhibited a decreasing trend following treatment with berberine, showing significant differences at a dosage of 100 mg/kg of berberine (*p* < 0.05). Additionally, *tnf-α* expression was notably suppressed in the group treated with 50 mg/kg of berberine compared with that in the HFD group (*p* < 0.05). 

### 3.7. Changes in the Expression of Genes Related to Immune Function

**Figure 7:**
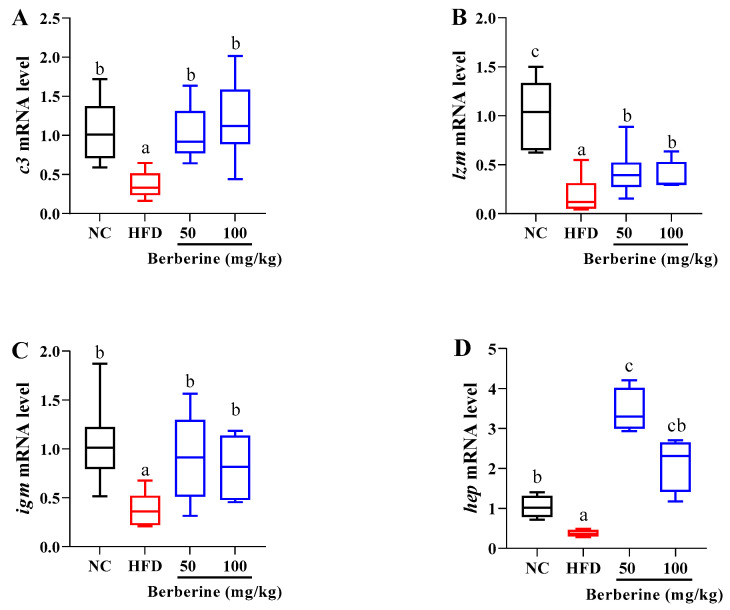
Relative expression of genes related to immune function in liver of tilapia fed berberine-inclusive high-fat diet. Different lowercase letters indicate significant differences between groups. (**A**) Complement C3 (*c3*). (**B**) Lysozyme C (*lzm*). (**C**) Immunoglobulin (*igm*). (**D**) Hepcidin (*hep*).

The qPCR analysis revealed that in the liver of tilapia subjected solely to an HFD, there was a significant reduction in the transcript levels of *c3*, *lzm*, *igm*, and *hep*. However, this reduction was ameliorated under treatments with berberine at concentrations of 50 and 100 mg/kg (*p* < 0.05; Figure 7). 

## 4. Discussion

Berberine has been identified as a therapeutic agent for liver diseases, including both chronic and acute hepatic damage [16]. Earlier studies have highlighted its hepatoprotective properties, demonstrating that berberine mitigates CCl_4_-induced acute hepatotoxicity in rats [38]. An in vitro study has further confirmed berberine’s capability to protect hepatocytes from hypoxia/reoxygenation (H/R)-induced damage [39]. In fish, berberine has been shown to protect against chronic copper-induced liver injury in *A. fasciatus*, significantly diminishing the serum levels of GPT and GOT [24]. Consistent with these findings, our study revealed a marked elevation in the activities of GPT and GOT in tilapia subjected to an HFD alone, indicative of severe liver injury. Conversely, this injurious trend was notably reversed following berberine treatment with 50 and/or 100 mg/kg, where the levels of these markers were nearly normalized, indicating berberine’s efficacy in counteracting HFD-induced liver damage in fish.

### 4.1. Effects of Berberine on the Metabolism Function 

TC and TG are principal components of fish blood lipids, serving as critical markers for pathological diagnosis. They reflect the lipid metabolism of the liver and the overall health status of the fish. The elevations in plasma TC, TG, LDL-c, and HDL-c following an HFD indicate a significant disruption in lipid metabolism [40]. Specifically, the increase in LDL-c and TC are concerning, pointing to an elevated risk of fatty liver disease development, which can further compromise fish health through the enhancement of oxidative stress and inflammatory responses [41]. Although the increase in HDL-c is often deemed positive in health due to its role in facilitating the reverse transport of cholesterol, its simultaneous elevation with LDL-c and TG after an HFD feeding may suggest an incapacity in the lipid regulatory system in fish [42]. In this study, the ability of berberine (50 and/or 100 mg/kg) to significantly reduce LDL-c, TC, and TG levels in tilapia highlighted its comprehensive effectiveness in ameliorating disruptions in lipid metabolism. Similar results were also reported in other fish species. For instance, dietary berberine decreased serum levels of LDL-c, TC, and TG in *Pelteobagrus fulvidraco* [43]; similarly, it resulted in reductions in serum levels of LDL-c, HDL-c, TC, and TG in *Ctenopharyngodon idella* [44]. These findings suggest berberine’s potential beneficial impact in ameliorating lipid imbalances, highlighting its versatile role in aquaculture nutrition. Nonetheless, in *M. amblycephala*, dietary berberine failed to suppress the elevation of TG and TC induced by an HFD [23]. The observed variability in response across different species underscores the imperative for additional research to elucidate the underlying mechanisms of berberine’s effects.

In the liver, lipid accumulation is closely associated with fatty acid β-oxidation. The activity of rate-limiting enzymes for β-oxidation, such as CPT-1 and AOX-1, plays a significant role in this process. PPARα, in particular, is recognized as a central regulator of lipid metabolism. It activates specific target genes (e.g., *cpt-1 and acox-1*) to facilitate fatty acid transport, oxidation, and lipogenesis [45]. Activation of PPARα not only ameliorates the metabolic syndrome but also exhibits anti-inflammatory effects [46]. In HFD-fed fish, a significant consequence was the downregulation of *cpt-1*, *acox-1*, and *pparα*, leading to reduced β-oxidation [47]. This diminished β-oxidation signifies a dysregulation in hepatic fatty acids, which in turn accelerates lipid accumulation and induces liver injury. It has been reported that berberine can enhance fatty acid β-oxidation, leading to a reduction in lipid accumulation. For instance, in *M. piceus*, the inclusion of dietary berberine mitigated the suppression of *cpt-1*, *acox-1*, and *pparα* genes provoked by an HFD feeding [32]. Likewise, in *M. amblycephala*, there was a notable reduction in the expression of *cpt-1*, *pparα,* and *acox* genes after an HFD feeding; however, supplementation with berberine effectively reversed this downregulation [32,48]. In line with these findings, our study revealed that the expression levels of *cpt-1* and *pparα* genes were significantly lower in fish fed an HFD, but this downregulation was counteracted by 100 mg/kg of berberine supplementation. Additionally, the lower *pparα* expression was also ameliorated with 50 mg/kg of berberine. This suggests that berberine may alleviate liver lipid accumulation in tilapia by modulating β-oxidation through the PPARα pathway.

Fatty liver disease is associated not only with lipid metabolism but also with other metabolic functions. Glutamine synthase (GS) is pivotal in nitrogen metabolism, regulates the homeostasis of blood ammonium ions and glutamine, and contributes to the modulation of liver functions [49]. In the model of CCl_4_-induced liver damage, GS activity was observed to decrease [50]; similarly, in the case of HFD-induced metabolic disorders, the expression of hepatic GS was found to be downregulated [51]. This study also noted a reduction in *gs* expression following HFD feeding, yet this downregulation was not significantly reversed by berberine treatment, indicating that berberine may not effectively mitigate the abnormalities in glutamine metabolism caused by an HFD feeding. 

UDP-glucuronosyltransferases (UGTs) are essential phase II drug-metabolizing enzymes that facilitate the detoxification of various substances, whose dysregulation can lead to metabolic disorders and improper management of xenobiotics. In rats subjected to chronic CCl_4_-induced liver fibrosis, an alteration in the mRNA level of *ugt* isoforms was observed, with *ugt1a1, 1a6, 2b1,* and *2b2* mRNA levels being elevated, while *ugt2b3*, *2b6*, and *2b12* mRNA levels were found to be reduced [52]. Similarly, the progression of human non-alcoholic fatty liver disease (NAFLD) is accompanied by a selective upregulation of certain UGT isoforms, notably UGT1A1, UGT1A3, UGT2B10, and UGT1A9 [53]. In our study, the mRNA level of *ugt2a2* was upregulated in the HFD group; however, treatment with 100 mg/kg of berberine mitigated this upregulation. This finding suggests that berberine possesses the potential to ameliorate UGT dysregulation and consequently alleviate metabolic disturbances.

NADH-cytochrome b5 reductase (CBR), an integral membrane enzyme, plays a pivotal role in several biochemical processes linked to liver health and disease, including fatty acid metabolism, drug processing, and antioxidant function. It has reported that the activity of CBR showed a significant increase in rats with liver injury induced by CCl_4_ and HFD, while this augmentation is mitigated by hepatoprotective agents such as d-limonene, silymarin, and trans-anethole [54,55]. Consistent with these findings, an increase in the mRNA level of the *cbr2* was observed in tilapia with HFD-induced liver injury, which was, however, alleviated by 50 and 100 mg/kg of berberine treatments. This suggests that berberine may have a regulatory effect on detoxification processes, contributing to the mitigation of liver damage.

### 4.2. Effect of Berberine on Antioxidant Status

Berberine is known for its antioxidant activity and its capacity to scavenge free radicals. It mitigates oxidative stress, as demonstrated by the modulation of antioxidant enzyme activities and the levels of oxidative stress indicators, including GSH and MDA [56]. Berberine can enhance the levels of T-AOC, SOD, and catalase (CAT), while suppressing the formation of MDA in the liver of *Micropterus salmoides* [57]. Berberine also prevents the reduction of antioxidant activity and formation of lipid peroxidation induced by acetaminophen in the liver of *Cyprinus carpio* [58]. Similarly, our findings demonstrated the remarkable antioxidative properties of berberine (50 and/or 100 mg/kg); it effectively mitigated oxidative damage induced by HFD feeding, maintained the normal levels of SOD (50 and 100 mg/kg) and GSH (100 mg/kg), and inhibited the formation of MDA (50 and 100 mg/kg) in liver tissues. This aligns with previously published findings that demonstrated the concomitant administration of berberine in HFD-fed *M. amblycephala*, *I. punctatus*, and rats significantly mitigated the reduction of antioxidant components (e.g., SOD and GSH) and the elevation of lipid peroxidation [23,59,60].

In liver injury induced by oxidative stress, a variety of critical molecules and pathways have been identified. Among these, the Nrf2 pathway is particularly notable for its extensive research focus related to oxidative stress. Nrf2, a transcription factor, plays a crucial role in protecting against oxidative damage via enhancing the expression of various cellular antioxidant defense proteins, including HO-1, GSTα, and NQO1 [61]. Studies have consistently demonstrated the therapeutic and biological effects of berberine through the activation of the Nrf2 pathway [62]. Specifically, berberine has been shown to mitigate liver injury induced by methotrexate, HFD, and CCl_4_ through the activation of the Nrf2 pathway in rats [60,63,64]. In fish, it was also found that dietary berberine enhanced the antioxidant capacity by upregulating *nrf2* expression [57,65]. In this study, the *nrf2* expression was markedly downregulated in HFD-induced liver injury, but this downregulation was reversed to normal levels following treatments with 50 and 100 mg/kg of berberine. Correspondingly, the mRNA levels of its downstream antioxidant genes *ho-1* and *gstα* were significantly downregulated in the HFD-treated group. However, berberine treatment at doses of 50 and 100 mg/kg notably increased *gstα* expression, and the 50 mg/kg of berberine markedly elevated *ho-1* expression. These findings suggest that berberine’s antioxidative effects may be attributed not only to its ROS scavenging activity but also to its enhancement of detoxifying/antioxidant enzyme expression through the activation of the Nrf2 signaling pathway in the liver of tilapia.

### 4.3. Effect of Berberine on Inflammatory and Immune Response

TLR2, a pattern recognition receptor of the innate immune system, is essential in connecting inflammation and liver injury, especially through its regulation of inflammatory responses under HFD-induced liver damage [66]. It has been reported that TLR2 activation triggers MyD88, subsequently initiating the NF-κB pathway to modulate the inflammatory response [67]. Mice lacking TLR2 gene exhibited notable reductions in inflammation, steatosis, and the development of non-alcoholic steatohepatitis [68,69]. Meanwhile, TLR2/NF-κB pathway activation was observed in mice subjected to an HFD, contributing to vascular inflammation [70]. Our study also found similar results, as we noted significant upregulation of *tlr2*, *myd88*, and NF-κB (*relb*) mRNA levels in HFD-induced liver injury. Importantly, treatment with 100 mg/kg berberine significantly attenuated these upregulations, suggesting that berberine may mitigate inflammation through the modulation of the TLR2/Myd88/NF-kB pathway. This finding corroborates previous research results, demonstrating that the anti-inflammatory effects of berberine are associated with the TLR (including TLR2) pathway [71].

Activation of the TLR2/MyD88/NF-κB signaling pathway initiates a cascade of genes associated with inflammatory cytokines, such as IL-1β, IL-8, and TNF-α, thereby exacerbating hepatic injury [72]. Indeed, after HFD feeding, upregulation of pro-inflammatory cytokines in the livers of both mice and fish was observed [59,73]. Similarly, in this study, *il-1β*, *il-8*, and *tnf-α* exhibited high expression levels in the liver of tilapia subjected to HFD treatment. In contrast, these elevated expressions of *il-1β*, *il-8*, and *tnf-α* were significantly reduced following treatment with 100 mg/kg of berberine. These findings suggest that the protective effect of berberine on the liver may be attributed to its anti-inflammatory properties. Supporting this notion, Wang et al., (2022) found that berberine mitigated the highly expressed *il-1β*, *tnf-α,* and *nf-κB* in *A. schlegelii* treated with an HFD [33]. Moreover, berberine diminished inflammation and lowered the levels of TNF-α, IL-6, and IL-1β in rats with non-alcoholic fatty liver disease by regulating the TLR4/MyD88/NF-κB signaling pathway [74].

In addition to inflammation, a suppression of immunity emerged as another consequence of liver injury induced by an HFD [59]. In fish, several innate immune parameters, such as LZM, HEP, and C3, play a key role in protecting against pathogens and contributing to the overall defense mechanisms [75,76]. The reduced LZM activity was observed in the plasma of *C. carpio* fed an HFD [77]. Similarly, reductions in LZM and C3 were noted in the plasma of blunt snout bream fed an HFD [78]. Furthermore, an HFD led to the downregulation of these proteins in the liver of mice [79,80]. In addition, IgM, a key player in the primary immune response, was also found to be decreased in the plasma of *C. carpio* and *O. niloticus* after HFD feeding [77,81]. Our data aligned with those in these studies, demonstrating that HFD treatment led to the suppression of mRNA levels of *lzm*, *hep*, *c3*, and *igm* in the liver of tilapia. The immunostimulatory effects of berberine have been well-documented, evidenced by a significant increase in LZM activity in the plasma of *O. niloticus* [82], alongside elevated levels of IgM and C3 in the intestine of *A. schlegelii* [65]. In line with these findings, our data demonstrated that berberine treatments (50 and 100 mg/kg) markedly reversed the HFD-induced downregulation of *lzm, hep, c3*, and *igm*, suggesting that berberine exerted a positive effect on the immune function in the liver of tilapia.

## 5. Conclusions

**Figure 8:**
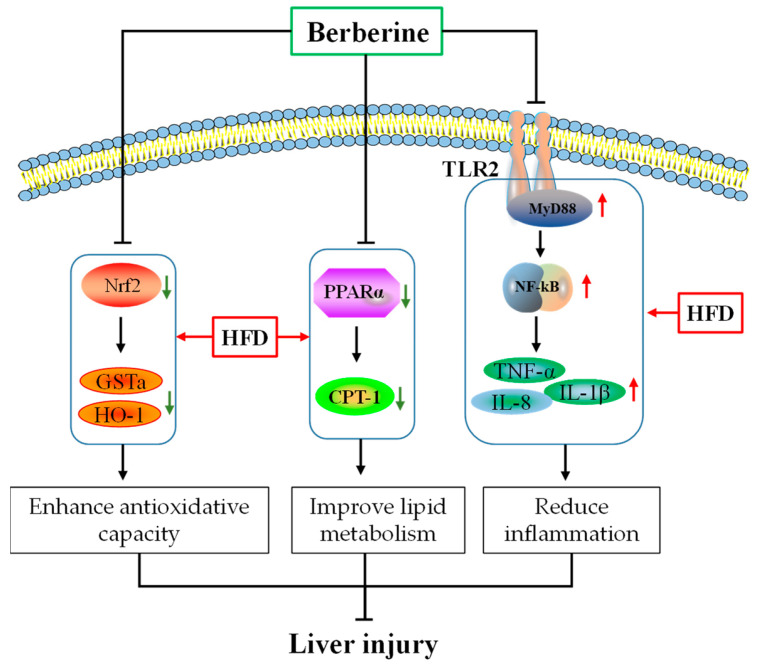
Possible mechanisms of berberine in ameliorating liver injury induced by HFD in tilapia. Red arrows indicate stimulatory modification, green arrows indicate inhibitory modification.

In summary, our findings indicated that berberine conferred hepatoprotective effects in tilapia by enhancing antioxidative and immune capacity, reducing inflammation, and improving lipid metabolism (Figure 8). The beneficial effects of berberine may be attributed to the enhancement of Nrf2 and PPARα signaling pathways, along with the inhibition of the TLR2/MyD88/NF-κB signaling pathway. Specifically, the upregulation of the Nrf2 pathway triggers the production of phase II detoxifying/antioxidant enzymes, such as HO-1 and GSTa, while the downregulation of the TLR2/MyD88/NF-κB pathway results in a decreased production of pro-inflammatory cytokines, including IL-1β, IL-8, and TNF-α. Additionally, increased PPARα is believed to enhance fatty acid β-oxidation, which alleviates liver lipid deposition.

## Figures and Tables

**Table 1:** The primer sequences used in the present study.

Gene	Primer Sequence (5′-3′)	GenBank Number/References
Nuclear factor erythroid 2-related factor 2 (*nrf2*)	F: CTGCCGTAAACGCAAGATGG	XM_003447296.5
	R: ATCCGTTGACTGCTGAAGGG	
NAD(P)H dehydrogenase 1 (*nqo1*)	F: TGGATTTCAGGTTCTGGCTCC	XM_013273094.3
	R: TCCTGTGGAGATGCCGAGA	
Glutathione S-transferase (*gsta*)	F: TAATGGGAGAGGGAAGATGG	NM_001279635.1
	R: CTCTGCGATGTAATTCAGGA	
Heme oxygenase (*ho-1*)	F: CTTGCCCGTGTGGAATCACT	XM_013270165.3
	R: AGATCACCGAGGTAGCGAGT	
Peroxisome proliferator activated receptor alpha (*pparα*)	F: CTGATAAAGCTTCGGGCTTCCA	NM_001290066.1
	R: CGCTCACACTTATCATACTCCAGCT	
Carnitine O-palmitoyltransferase 1 (*cpt-1*)	F: TTTCCAGGCCTCCTTACCCA	XM_013268638.3
	R: TGTACTGCTCATTGTCCAGCAGA	
Acyl-CoA oxidase 1 (*acox-1*)	F: GGTCAAAGGCAACAATCAGGAG	NM_001290199.1
	R: GACTCTGCCAAAGGCAACCA	
Toll-like receptor 2 (*tlr2*)	F: AAAAGCATAGATGAGTTCCACATCC	JQ809459.1
	R: GTAAGACAAGGCATCACAAACACC	
Myloid differentiation factor 88 (*myd88*)	F: CAGGTTCCTGAGGTCGACAG	KJ130039.1
	R: CATTTCGTGGACGAACGCAA	
NF-kB subunit (*relb*)	F: TCACTGCCTCCACCTTTGCT	XM_005459330.4
	R: ATCCTCATAGTTCCTCTTCCGTTTT	
Tumor necrosis factor-alpha (*tnf-α*)	F: AAGCCAAGGCAGCCATCCAT	[35]
	R: TTGACCATTCCTCCACTCCAGA	
Interleukin-1 beta (*il-1β*)	F: TCAGTTCACCAGCAGGGATG	[36]
	R: GACAGATAGAGGTTTGTGCC	
Interleukin-8 (*il-8*)	F: CTGTGAAGGCATGGGTGTGGAG	[35]
	R: TCGCAGTGGGAGTTGGGAAGAA	
Glutamine synthase a (*gs*)	F: AGCTACCACATTCGTGCCTAC	NM_001279668.1
	R: TACGAGGAATGCGAATGCTGG	
UDP-glucuronosyltransferase 2A2 (*ugt2a2*)	F: GGTGCTGTGTCAGGAAAGGAA	XM_025896953.1
	R: ATCAAACTAGCCACCTTTGGCA	
NADH-cytochrome b5 reductase 2 (*cbr2*)	F: ATCGCTGGTGGAACAGGTATC	XM_003439423.3
	R:TGTGGAGGTTTGTCCAGTGT	
Lysozyme C (*lzm*)	F: AAGGGAAGCAGCAGCAGTTGTG	XM_019361339.1
	R: CGTCCATGCCGTTAGCCTTGAG	
Immunoglobulin *(igm)*	F: ACCGAATCGAAAAATGCGGC	KJ676389.1
	R: AACACAACCAGGACATTGGTTC	
Complement C3 (*c3*)	F: GGTGTGGATGCACCTGAGAA	XM_013274267.3
	R: GGGAAATCGGTACTTGGCCT	
Hepcidin (*hep*)	F: GACACAAGCGTGGCATCAAG	XM_019365122.2
	R: GTTGAGGCAGTAACTGAGGACA	
Ubiquitin-conjugating enzyme (*ubce*)	F: CTCTCAAATCAATGCCACTTCC	[37]
	R: CCCTGGTGGAGGTTCCTTGT	

## Data Availability

All data are contained within the main manuscript.

## References

[1] de Lima-Faria J.M., da Silva V.C., Chen L.C., Martinez D.S.T., de Sabóia-Morais S.M.T. (2023). Co-exposure of iron oxide nanoparticles with glyphosate herbicides in Poecilia reticulata: Fish liver damages is reversible during iron accumulation and elimination period. Chemosphere.

[2] Wolf J.C., Wolfe M.J. (2005). A brief overview of nonneoplastic hepatic toxicity in fish. Toxicol. Pathol..

[3] Wang T., Wei X., Chen T., Wang W., Xia X., Miao J., Yin S. (2019). Studies of the mechanism of fatty liver formation in Takifugu fasciatus following copper exposure. Ecotoxicol. Environ. Saf..

[4] Wang X., Li Y., Hou C., Gao Y., Wang Y. (2015). Physiological and molecular changes in large yellow croaker (P seudosciaena crocea R.) with high-fat diet-induced fatty liver disease. Aquac. Res..

[5] Schlegel A. (2012). Studying non-alcoholic fatty liver disease with zebrafish: A confluence of optics, genetics, and physiology. Cell. Mol. Life Sci..

[6] Asaoka Y., Terai S., Sakaida I., Nishina H. (2013). The expanding role of fish models in understandingnon-alcoholic fatty liver disease. Dis. Models Mech..

[7] Tao Y.-F., Qiang J., Bao J.-W., Chen D.-J., Yin G.-J., Xu P., Zhu H.-J. (2018). Changes in Physiological Parameters, Lipid Metabolism, and Expression of MicroRNAs in Genetically Improved Farmed Tilapia (*Oreochromis niloticus*) With Fatty Liver Induced by a High-Fat Diet. Front. Physiol..

[8] Qiang J., He J., Yang H., Sun Y.-L., Tao Y.-F., Xu P., Zhu Z.-X. (2017). Dietary lipid requirements of larval genetically improved farmed tilapia, *Oreochromis niloticus* (L.), and effects on growth performance, expression of digestive enzyme genes, and immune response. Aquac. Res..

[9] Dai Y.-J., Cao X.-F., Zhang D.-D., Li X.-F., Liu W.-B., Jiang G.-Z. (2019). Chronic inflammation is a key to inducing liver injury in blunt snout bream (*Megalobrama amblycephala*) fed with high-fat diet. Dev. Comp. Immunol..

[10] Cao X.-F., Dai Y.-J., Liu M.-Y., Yuan X.-Y., Wang C.-C., Huang Y.-Y., Liu W.-B., Jiang G.-Z. (2019). High-fat diet induces aberrant hepatic lipid secretion in blunt snout bream by activating endoplasmic reticulum stress-associated IRE1/XBP1 pathway. Biochim. Biophys. Acta BBA Mol. Cell Biol. Lipids.

[11] Du Z. (2014). Causes of fatty liver in farmed fish: A review and new perspectives. J. Fish. China.

[12] Zhang F.-L., Hao Q., Zhang Q.-S., Lv H.-Y., Yang Y.-L., Chao R., Zhang Z., Zhou Z.-G. (2022). Influences of dietary Eucommia ulmoides leaf extract on the hepatic lipid metabolism, inflammation response, intestinal antioxidant capacity, intestinal microbiota, and disease resistance of the channel catfish (*Ictalurus punctatus*). Fish Shellfish Immunol..

[13] Zheng Y., Shi Y., Yang X., Gao J., Nie Z., Xu G. (2022). Effects of resveratrol on lipid metabolism in liver of red tilapia Oreochromis niloticus. Comp. Biochem. Physiol. Part C Toxicol. Pharmacol..

[14] Zou C.Y., Du L.K., Wu J.H., Gan S.Y., Li Q.Q., Babu V.S., Wu Y.X., Lin L. (2022). Saikosaponin d alleviates high-fat-diet induced hepatic steatosis in hybrid grouper (*Epinephelus lanceolatusd*♂ × *Epinephelus fuscoguttatus*♀) by targeting AMPK/PPARα pathway. Aquaculture.

[15] Jin M., Shen Y.D., Pan T.T., Zhu T.T., Li X.J., Xu F.M., Betancor M.B., Jiao L.F., Tocher D.R., Zhou Q.C. (2021). Dietary Betaine Mitigates Hepatic Steatosis and Inflammation Induced by a High-Fat-Diet by Modulating the Sirt1/Srebp-1/Pparalpha Pathway in Juvenile Black Seabream (*Acanthopagrus schlegelii*). Front. Immunol..

[16] Zhou M., Deng Y., Liu M., Liao L., Dai X., Guo C., Zhao X., He L., Peng C., Li Y. (2021). The pharmacological activity of berberine, a review for liver protection. Eur. J. Pharmacol..

[17] Kavyani Z., Shahhosseini E., Moridpour A.H., Falahatzadeh M., Vajdi M., Musazadeh V., Askari G. (2023). The effect of berberine supplementation on lipid profile and obesity indices: An umbrella review of meta-analysis. PharmaNutrition.

[18] Gaba S., Saini A., Singh G., Monga V. (2021). An insight into the medicinal attributes of berberine derivatives: A review. Bioorg. Med. Chem..

[19] Wang L., Sagada G., Wang C., Gao C., Wang B., Shao Q., Yan Y. (2022). Berberine in fish nutrition: Impact on hepatoenteric health, antioxidative and immune status. Front. Mar. Sci..

[20] Xu W.-N., Chen D.-H., Chen Q.-Q., Liu W.-B. (2017). Growth performance, innate immune responses and disease resistance of fingerling blunt snout bream, Megalobrama amblycephala adapted to different berberine-dietary feeding modes. Fish Shellfish Immunol..

[21] Yu C.B., Zhang J., Qin Q., Liu J., Xu J.X., Xu W.N. (2020). Berberine improved intestinal barrier function by modulating the intestinal microbiota in blunt snout bream (*Megalobrama amblycephala*) under dietary high-fat and high-carbohydrate stress. Fish Shellfish Immunol..

[22] Ming J.H., Wang T., Wang T.H., Ye J.Y., Zhang Y.X., Yang X., Shao X.P., Ding Z.Y. (2023). Effects of dietary berberine on growth performance, lipid metabolism, antioxidant capacity and lipometabolism-related genes expression of AMPK signaling pathway in juvenile black carp (*Mylopharyngodon piceus*) fed high-fat diets. Fish Physiol. Biochem..

[23] Lu K.-L., Wang L.-N., Zhang D.-D., Liu W.-B., Xu W.-N. (2017). Berberine attenuates oxidative stress and hepatocytes apoptosis via protecting mitochondria in blunt snout bream *Megalobrama amblycephala* fed high-fat diets. Fish Physiol. Biochem..

[24] Wang C., Wang L., Yang L., Gao C., Wang B., Shu Y., Wang H., Yan Y. (2023). Protective effects of berberine in chronic copper-induced liver and gill injury in freshwater grouper (*Acrossocheilus fasciatus*). Ecotoxicol. Environ. Saf..

[25] Xu F., Xu C., Xiao S., Lu M., Limbu S.M., Wang X., Du Z., Qin J.G., Chen L. (2019). Effects of α-lipoic acid on growth performance, body composition, antioxidant profile and lipid metabolism of the GIFT tilapia (Oreochromis niloticus) fed high-fat diets. Aquac. Nutr..

[26] Lin J.-J., Liu Y.-C., Chang C.-J., Pan M.-H., Lee M.-F., Pan B.S. (2018). Hepatoprotective mechanism of freshwater clam extract alleviates non-alcoholic fatty liver disease: Elucidated in vitro and in vivo models. Food Funct..

[27] Qiang J., Tao Y.F., Bao J.W., Chen D.J., Li H.X., He J., Xu P. (2018). High Fat Diet-Induced miR-122 Regulates Lipid Metabolism and Fat Deposition in Genetically Improved Farmed Tilapia (GIFT, *Oreochromis niloticus*) Liver. Front. Physiol..

[28] Yu K., Huang K., Tang Z., Huang X., Sun L., Pang L., Mo C. (2021). Metabolism and antioxidation regulation of total flavanones from *Sedum sarmentosum* Bunge against high-fat diet-induced fatty liver disease in Nile tilapia (*Oreochromis niloticus*). Fish Physiol. Biochem..

[29] Du J., Jia R., Cao L., Gu Z., He Q., Xu P., Yin G., Ma Y. (2022). Regulatory effects of *Glycyrrhiza* total flavones on fatty liver injury induced by a high-fat diet in tilapia (*Oreochromis niloticus*) via the Nrf2 and TLR signaling pathways. Aquac. Int..

[30] Sheng C.H., Jing J.L., Lee M.F., Liu Y.C., Pan B.S. (2016). Freshwater clam extracts alleviate dyslipidaemia of tilapia fed a high-fat diet as an animal model. J. Funct. Foods.

[31] He A.Y., Ning L.J., Chen L.Q., Chen Y.L., Xing Q., Li J.M., Qiao F., Li D.L., Zhang M.L., Du Z.Y. (2015). Systemic adaptation of lipid metabolism in response to low- and high-fat diet in Nile tilapia (*Oreochromis niloticus*). Physiol. Rep..

[32] Zhou W.H., Rahimnejad S., Lu K.L., Wang L.N., Liu W.B. (2019). Effects of berberine on growth, liver histology, and expression of lipid-related genes in blunt snout bream (*Megalobrama amblycephala*) fed high-fat diets. Fish Physiol. Biochem..

[33] Wang L., Sagada G., Xu B., Zhang J., Shao Q. (2022). Influence of dietary berberine on liver immune response and intestinal health of black sea bream (*Acanthopagrus schlegelii*) fed with normal and high-lipid diets. Aquac. Nutr..

[34] Livak K., Schmittgen T. (2001). Analysis of relative gene expression data using real-time quantitative PCR and the 2^−∆∆CT^ Method. Methods-Companion Methods Enzymol..

[35] Limbu S.M., Zhou L., Sun S.X., Zhang M.L., Du Z.Y. (2018). Chronic exposure to low environmental concentrations and legal aquaculture doses of antibiotics cause systemic adverse effects in Nile tilapia and provoke differential human health risk. Environ. Int..

[36] Ken C.F., Chen C.N., Ting C.H., Pan C.Y., Chen J.Y. (2017). Transcriptome analysis of hybrid tilapia (*Oreochromis* spp.) with Streptococcus agalactiae infection identifies Toll-like receptor pathway-mediated induction of NADPH oxidase complex and piscidins as primary immune-related responses. Fish Shellfish Immunol..

[37] Chang G.Y., Xian L.W., Tian J., Wei L., Fan W., Ming J., Hua W. (2013). Evaluation of reference genes for quantitative real-time RT-PCR analysis of gene expression in Nile tilapia (*Oreochromis niloticus*). Gene.

[38] Feng Y., Siu K.-Y., Ye X., Wang N., Yuen M.-F., Leung C.-H., Tong Y., Kobayashi S. (2010). Hepatoprotective effects of berberine on carbon tetrachloride-induced acute hepatotoxicity in rats. Chin. Med..

[39] Zhu Y., Li J., Zhang P., Peng B., Li C., Ming Y., Liu H. (2023). Berberine protects hepatocyte from hypoxia/reoxygenation-induced injury through inhibiting circDNTTIP2. PeerJ.

[40] Gong X., Li T., Wan R., Sha L. (2021). Cordycepin attenuates high-fat diet-induced non-alcoholic fatty liver disease via down-regulation of lipid metabolism and inflammatory responses. Int. Immunopharmacol..

[41] Yang B., Shen Y., Monroig Ó., Zhao W., Bao Y., Tao S., Jiao L., Zhou Q., Jin M. (2024). The ameliorative role of methionine in hepatic steatosis and stress response in juvenile black seabream (*Acanthopagrus schlegelii*) fed with a high-fat diet. Aquaculture.

[42] Barter P. (2011). HDL-C: Role as a risk modifier. Atheroscler. Suppl..

[43] Wang L., Gao C., Yang L., Wang C., Wang B., Wang H., Shu Y., Yan Y. (2023). The growth-promoting and lipid-lowering effects of berberine are associated with the regulation of intestinal bacteria and bile acid profiles in yellow catfish (*Pelteobagrus fulvidraco*). Aquac. Rep..

[44] Tian J.-J., Jin Y.-Q., Yu E.-M., Sun J.-H., Xia Y., Zhang K., Li Z.-F., Gong W.-B., Wang G.-J., Xie J. (2022). Intestinal farnesoid X receptor mediates the effect of dietary berberine on lipid accumulation in grass carp (*Ctenopharyngodon idella*). Aquaculture.

[45] Wang Y.P., Nakajima T., Gonzalez F.J., Tanaka N. (2020). PPARs as Metabolic Regulators in the Liver: Lessons from Liver-Specific PPAR-Null Mice. Int. J. Mol. Sci..

[46] Yang Z., Roth K., Agarwal M., Liu W., Petriello M.C. (2021). The transcription factors CREBH, PPARa, and FOXO1 as critical hepatic mediators of diet-induced metabolic dysregulation. J. Nutr. Biochem..

[47] Jia R., Cao L.-P., Du J.-L., He Q., Gu Z.-Y., Jeney G., Xu P., Yin G.-J. (2020). Effects of high-fat diet on steatosis, endoplasmic reticulum stress and autophagy in liver of tilapia (*Oreochromis niloticus*). Front. Mar. Sci..

[48] Lu K.-L., Zhang D.-D., Wang L.-N., Xu W.-N., Liu W.-B. (2016). Molecular characterization of carnitine palmitoyltransferase IA in Megalobrama amblycephala and effects on its expression of feeding status and dietary lipid and berberine. Comp. Biochem. Physiol. Part B Biochem. Mol. Biol..

[49] Frieg B., Görg B., Gohlke H., Häussinger D. (2021). Glutamine synthetase as a central element in hepatic glutamine and ammonia metabolism: Novel aspects. Biol. Chem..

[50] Savilov P.N., Yakovlev V.N. (2016). Effect of Liver Damage and Hyperbaric Oxygenation on Glutamine Synthetase of Hepatocytes. Bull. Exp. Biol. Med..

[51] Fan Z., Wang S., Meng Y., Wen C., Xu M., Li X. (2023). Butyrate Alleviates High-Fat-Induced Metabolic Disorders Partially through Increasing Systematic Glutamine. J. Agric. Food Chem..

[52] Xu L., Zheng R., Xie P., Guo Q., Ji H., Li T. (2020). Dysregulation of UDP-glucuronosyltransferases in CCl4 induced liver injury rats. Chem. Biol. Interact..

[53] Hardwick R.N., Ferreira D.W., More V.R., Lake A.D., Lu Z., Manautou J.E., Slitt A.L., Cherrington N.J. (2013). Altered UDP-glucuronosyltransferase and sulfotransferase expression and function during progressive stages of human nonalcoholic fatty liver disease. Drug Metab. Dispos..

[54] Pandit K., Kumar A., Kaur S., Kumar V., Jain S.K., Bhardwaj R., Kaur S. (2022). Amelioration of oxidative stress by *trans*-Anethole via modulating phase I and phase II enzymes against hepatic damage induced by CCl_4_ in male Wistar rats. Environ. Sci. Pollut. Res. Int..

[55] Victor Antony Santiago J., Jayachitra J., Shenbagam M., Nalini N. (2012). Dietary d-limonene alleviates insulin resistance and oxidative stress-induced liver injury in high-fat diet and L-NAME-treated rats. Eur. J. Nutr..

[56] Ma X., Chen Z., Wang L., Wang G., Wang Z., Dong X., Wen B., Zhang Z. (2018). The pathogenesis of diabetes mellitus by oxidative stress and inflammation: Its inhibition by berberine. Front. Pharmacol..

[57] Chen S., Jiang X., Liu N., Ren M., Wang Z., Li M., Chen N., Li S. (2022). Effects of dietary berberine hydrochloride inclusion on growth, antioxidant capacity, glucose metabolism and intestinal microbiome of largemouth bass (*Micropterus salmoides*). Aquaculture.

[58] Grădinariu L., Dediu L., Crețu M., Grecu I.R., Docan A., Istrati D.I., Dima F.M., Stroe M.D., Vizireanu C. (2024). The Antioxidant and Hepatoprotective Potential of Berberine and Silymarin on Acetaminophen Induced Toxicity in *Cyprinus carpio* L.. Animals.

[59] Desouky H.E., Jiang G.-z., Abasubong K.P., Dai Y.-J., Yuan X., Adjoumani J.-J.Y., Liu W.-b. (2023). Plant-Based Additivities Improved the Growth Performance and Immune Response, and Mitigated the Inflammatory Signalling in Channel Catfish Fed a High-Fat Diet. Aquac. Res..

[60] Deng Y., Tang K., Chen R., Nie H., Liang S., Zhang J., Zhang Y., Yang Q. (2019). Berberine attenuates hepatic oxidative stress in rats with non-alcoholic fatty liver disease via the Nrf2/ARE signalling pathway. Exp. Ther. Med..

[61] Huang Y., Li W., Su Z.-y., Kong A.-N.T. (2015). The complexity of the Nrf2 pathway: Beyond the antioxidant response. J. Nutr. Biochem..

[62] Ashrafizadeh M., Fekri H.S., Ahmadi Z., Farkhondeh T., Samarghandian S. (2020). Therapeutic and biological activities of berberine: The involvement of Nrf2 signaling pathway. J. Cell. Biochem..

[63] Mahmoud A.M., Hozayen W.G., Ramadan S.M. (2017). Berberine ameliorates methotrexate-induced liver injury by activating Nrf2/HO-1 pathway and PPARγ, and suppressing oxidative stress and apoptosis in rats. Biomed. Pharmacother..

[64] Han C.Y., Sun T.T., Xv G.P., Wang S.S., Gu J.G., Liu C.Y. (2019). Berberine ameliorates CCl4-induced liver injury in rats through regulation of the Nrf2-Keap1-ARE and p53 signaling pathways. Mol. Med. Rep..

[65] Sagada G., Wang L., Xu B., Tegomo F.A., Chen K., Zheng L., Sun Y., Liu Y., Yang Y., Ullah S. (2022). Synergistic Effect of Dietary Inactivated Lactobacillus plantarum and Berberine Supplementation on Growth Performance, Antioxidant Capacity, and Immune Function of Juvenile Black Sea Bream (*Acanthopagrus schlegelii*). Aquac. Nutr..

[66] Khanmohammadi S., Kuchay M.S. (2022). Toll-like receptors and metabolic (dysfunction)-associated fatty liver disease. Pharmacol. Res..

[67] Ma J.Q., Li Z., Xie W.R., Liu C.M., Liu S.S. (2015). Quercetin protects mouse liver against CCl₄-induced inflammation by the TLR2/4 and MAPK/NF-κB pathway. Int. Immunopharmacol..

[68] Liu J., Zhuang Z.J., Bian D.X., Ma X.J., Xun Y.H., Yang W.J., Luo Y., Liu Y.L., Jia L., Wang Y. (2014). Toll-like receptor-4 signalling in the progression of non-alcoholic fatty liver disease induced by high-fat and high-fructose diet in mice. Clin. Exp. Pharmacol. Physiol..

[69] Miura K., Yang L., van Rooijen N., Brenner D.A., Ohnishi H., Seki E. (2013). Toll-like receptor 2 and palmitic acid cooperatively contribute to the development of nonalcoholic steatohepatitis through inflammasome activation in mice. Hepatology.

[70] Jang H.-J., Kim H.-S., Hwang D.H., Quon M.J., Kim J.-A. (2013). Toll-like receptor 2 mediates high-fat diet-induced impairment of vasodilator actions of insulin. Am. J. Physiol. Endocrinol. Metab..

[71] Wang X.-P., Lei F., Du F., Chai Y.-S., Jiang J.-F., Wang Y.-G., Yu X., Yan X.-J., Xing D.-M., Du L.-J. (2015). Protection of gastrointestinal mucosa from acute heavy alcohol consumption: The effect of berberine and its correlation with TLR2, 4/IL1β-TNFα signaling. PLoS ONE.

[72] Shan J.-L., Wei R.-R., Lu W., Ouyang X., Cheng H.-Y., Zhong G.-Y., Liu J.-C., Zhu J.-X. (2020). Mechanism of anti-chronic alcoholic liver injury in rats of tibetan medicine Lagotis brachystachys extracts by TLR2/MyD88/NF-κB and NALP3 signaling pathway. Chin. J. Exp. Tradit. Med. Formulae.

[73] Ghezelbash B., Shahrokhi N., Khaksari M., Asadikaram G., Shahrokhi M., Shirazpour S. (2022). Protective Roles of Shilajit in Modulating Resistin, Adiponectin, and Cytokines in Rats with Non-alcoholic Fatty Liver Disease. Chin. J. Integr. Med..

[74] Wang L., Jia Z., Wang B., Zhang B. (2020). Berberine inhibits liver damage in rats with non-alcoholic fatty liver disease by regulating TLR4/MyD88/NF-κB pathway. Turk. J. Gastroenterol..

[75] Shailesh S., Sahoo P.K. (2008). Lysozyme: An important defence molecule of fish innate immune system. Aquac. Res..

[76] Bao B., Peatman E., Li P., He C., Liu Z. (2005). Catfish hepcidin gene is expressed in a wide range of tissues and exhibits tissue-specific upregulation after bacterial infection. Dev. Comp. Immunol..

[77] Abasubong K.P., Li X.F., Adjoumani J.J.Y., Jiang G.Z., Desouky H.E., Liu W.B. (2022). Effects of dietary xylooligosaccharide prebiotic supplementation on growth, antioxidant and intestinal immune-related genes expression in common carp Cyprinus carpio fed a high-fat diet. J. Anim. Physiol. Anim. Nutr..

[78] Abasubong K.P., Jiang G.-Z., Guo H.-X., Wang X., Huang Y.-Y., Dai Y.-J., Li X.-F., Dong Y.-Z., Gabriel N.N., Liu W.-B. (2023). Oral bovine serum albumin administration alleviates inflammatory signals and improves antioxidant capacity and immune response under thioacetamide stress in blunt snout bream fed a high-calorie diet. Fish Shellfish Immunol..

[79] Padda R.S., Gkouvatsos K., Guido M., Mui J., Vali H., Pantopoulos K. (2015). A high-fat diet modulates iron metabolism but does not promote liver fibrosis in hemochromatotic Hjv^-/-^ mice. Am. J. Physiol.-Gastrointest. Liver Physiol..

[80] Li Y., Jiang W., Feng Y., Wu L., Jia Y., Zhao R. (2022). Betaine Alleviates High-Fat Diet-Induced Disruption of Hepatic Lipid and Iron Homeostasis in Mice. Int. J. Mol. Sci..

[81] Qian Y.-C., Wang X., Ren J., Wang J., Limbu S.M., Li R.-X., Zhou W.-H., Qiao F., Zhang M.-L., Du Z.-Y. (2021). Different effects of two dietary levels of tea polyphenols on the lipid deposition, immunity and antioxidant capacity of juvenile GIFT tilapia (*Oreochromis niloticus*) fed a high-fat diet. Aquaculture.

[82] Doan H.V., Hoseinifar S.H., Jaturasitha S., Dawood M.A.O., Harikrishnan R. (2020). The effects of berberine powder supplementation on growth performance, skin mucus immune response, serum immunity, and disease resistance of Nile tilapia (*Oreochromis niloticus*) fingerlings. Aquaculture.

